# Electrolyte Tuning in Iron(II)-Based Dye-Sensitized Solar Cells: Different Ionic Liquids and I_2_ Concentrations

**DOI:** 10.3390/ma14113053

**Published:** 2021-06-03

**Authors:** Mariia Becker, Catherine E. Housecroft, Edwin C. Constable

**Affiliations:** Department of Chemistry, University of Basel, BPR 1096, Mattenstrasse 24a, CH-4058 Basel, Switzerland; mariia.karpacheva@unibas.ch (M.B.); catherine.housecroft@unibas.ch (C.E.H.)

**Keywords:** *N*-heterocyclic carbene, iron(II), dye-sensitized solar cell, ionic liquid, iodine

## Abstract

The effects of different I_2_ concentrations and different ionic liquids (ILs) in the electrolyte on the performances of dye-sensitized solar cells (DSCs) containing an iron(II) *N*-heterocyclic carbene dye and containing the I^–^/I_3_^–^ redox shuttle have been investigated. Either no I_2_ was added to the electrolyte, or the initial I_2_ concentrations were 0.02, 0.05, 0.10, and 0.20 M. The short-circuit current density (*J*_SC_), open-circuit voltage (*V*_OC_), and the fill factor (*ff*) were influenced by changes in the I_2_ concentration for all the ILs. For 1-hexyl-3-methylimidazole iodide (HMII), low *V*_OC_ and low *ff* values led to poor DSC performances. Electrochemical impedance spectroscopy (EIS) showed the causes to be increased electrolyte diffusion resistance and charge transfer resistance at the counter electrode. DSCs containing 1,3-dimethylimidazole iodide (DMII) and 1-ethyl-3-methylimidazole iodide (EMII) showed the highest *J*_SC_ values when 0.10 M I_2_ was present initially. Short alkyl substituents (Me and Et) were more beneficial than longer chains. The lowest values of the transport resistance in the photoanode semiconductor were found for DMII, EMII, and 1-propyl-2,3-dimethylimidazole iodide (PDMII) when no I_2_ was added to the initial electrolyte, or when [I_2_] was less than 0.05 M. Higher [I_2_] led to decreases in the diffusion resistance in the electrolyte and the counter electrode resistance. The electron lifetime and diffusion length depended upon the [I_2_]. Overall, DMII was the most beneficial IL. A combination of DMII and 0.1 M I_2_ in the electrolyte produced the best performing DSCs with an average maximum photoconversion efficiency of 0.65% for a series of fully-masked cells.

## 1. Introduction

**Figure 1:**
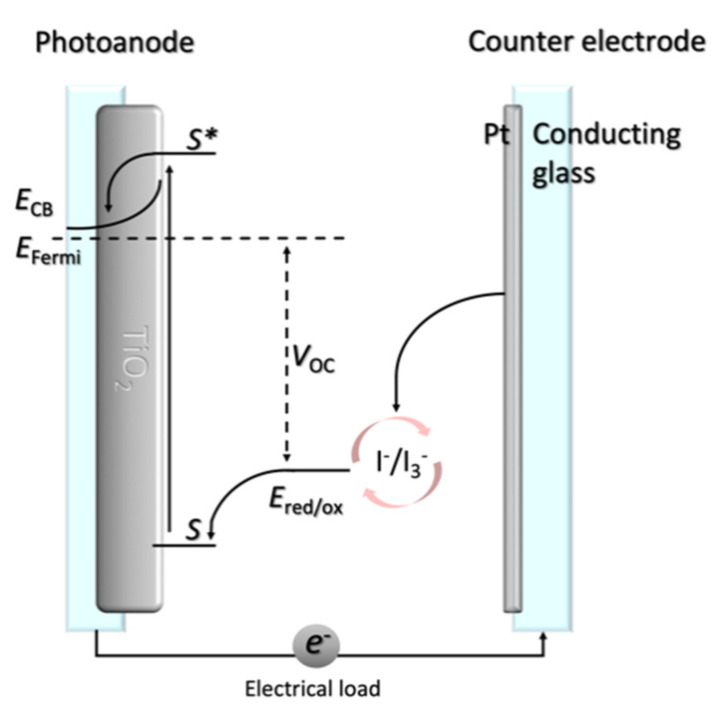
Schematic diagram of an *n*-type DSC. *S* and S* = ground and excited states of the dye; *V*_OC_ = open-circuit voltage; *E*_CB_ = energy of conduction band of the semiconductor; *E*_F_ = Fermi level; *E*_redox_ = redox potential of the redox shuttle.

Sustainable approaches to energy generation are of critical societal importance and are one of the United Nations Sustainable Development Goals (SDG7) [1]. The conversion of solar to electrical energy is a critical sustainable goal, and an alternative strategy to the well-established silicon photovoltaics is seen in the development of dye-sensitized solar cells (DSCs) [2]. An *n*-type DSC (Figure 1) employs a wide-bandgap *n*-type semiconductor, typically TiO_2_ (anatase), with a sensitizer adsorbed on the surface to extend the absorption of light into the visible region [3,4,5,6]. Sensitizers in DSCs are typically metal-free (organic) or ruthenium(II)-based dyes. Although state-of-the-art ruthenium dyes reach up to ca. 12% photoconversion efficiency (PCE or *η*) [7], the use of metals with low natural abundances does not address the problem of sustainability [8,9]. Thus, investigations of coordination compounds of Earth abundant metals, such as copper [10,11,12,13,14] and iron [14,15,16,17,18], as sensitizers in DSCs are of prime interest.

**Scheme 1:**
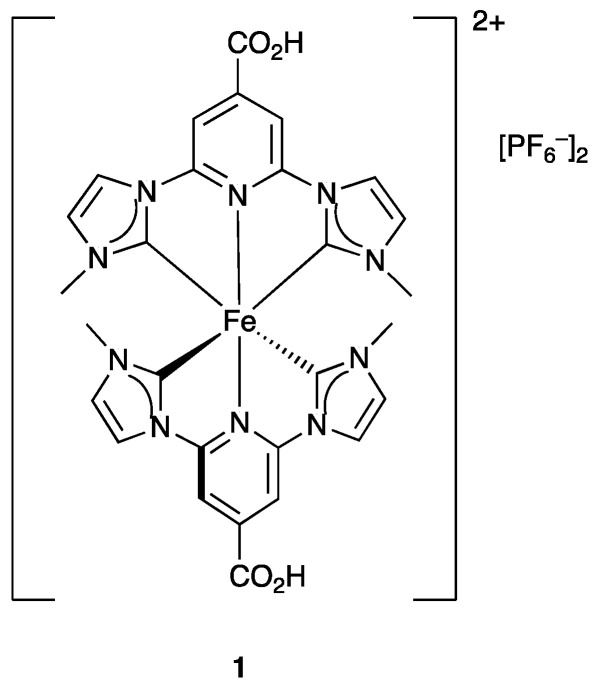
The structure of the iron(II)-NHC dye **1**.

The first DSCs based on [Fe(dcbpy)_2_(CN)_2_] (dcbpy = 2,2’-bipyridine-4,4’-dicarboxylic acid) were disappointing and produced only a very low short-circuit current density (*J*_SC_) and open-circuit voltage (*V*_OC_) [19]. In order to successfully utilize iron(II) complexes as sensitizers, one must overcome the problem of fast deactivation from a metal-to-ligand charge transfer (MLCT) state to a metal-centred (MC) state [16]. This deactivation leads to inefficient electron injection into the semiconductor and, therefore, low *J*_SC_ values. Tuning the electrolyte has been highly beneficial in enhancing the performance of [Fe(dcbpy)_2_(CN)_2_], and Jakubikova, McCusker and coworkers systematically increased values of *J*_SC_ from 0.10 to 1.46 mA cm^–2^ and η from 0.024% to 0.35% for masked DSCs by the addition of different salts and pyridine derivatives [20]. Cyclometallated iron(II) complexes have been suggested as one means of accessing more efficient iron-based photosensitizers [21]. However, a move to *N*-heterocyclic carbene (NHC) ligands proved to be the critical turning point in utilizing iron(II)-based dyes. After a report by Wärnmark and coworkers that the iron(II) NHC complex **1** (Scheme 1) exhibited a long ^3^MLCT lifetime of 9 ps [22], the group of Gros demonstrated that **1** performed in *n*-type DSCs with η = 0.13% [23]. Until very recently, complex **1** continued to be one of the most promising iron(II)-based sensitizers [15,24], and tuning the composition of the electrolyte has been crucial in improving the performance of DSCs sensitized by **1** [25,26]. Gros reported in 2020 that the presence of both Mg^2+^ ions and guanidium thiocyanate in the electrolyte with an I^–^/I_3_^–^ redox shuttle resulted in a high *J*_SC_ of 3.3 mA cm^–2^ and η of 1%. A critical factor to the improvement in DSC performance was the presence of an additional blocking underlayer on the electrodes [26]. While our own investigations for the current work were in progress, Gros and coworkers reported a series of heteroleptic iron(II) NHC dyes and achieved a value of η = 1.44% for masked devices, thereby setting a new record for Fe(II)-based DSCs [27]. Manipulation of the electrolyte composition (specifically, the addition of Mg^2+^ ions and Bu_4_NI), the use of poly(3,4-ethylenedioxythiophene (PEDOT) coated counter electrodes, and the application of a blocking underlayer on the electrodes proved to be important factors.

The I^–^/I_3_^–^ redox couple has been used for all investigations of iron(II)-NHC dyes reported to date. For DSCs containing other sensitizers, it has been established that additional I*_n_*^–^ species are formed in the electrolyte at high I_2_ concentrations but that they are not of importance in the device [28]. The operation of a DSC is based on effective dye regeneration after electron injection into conduction band of the semiconductor, and this is a multistep process. After dye excitation, the dye is formally in its oxidized state (D^+^), and this is reduced by I^–^ ion from the redox shuttle after initial formation of a dye-iodide complex, D^+^:I^–^. The formation of this complex has been studied for [Ru(dcbpy)_2_X_2_], and is considered to be key for dye regeneration [29]. The addition of a second iodide ion results in complex dissociation and formation of the radical anion I_2_^–^ and the dye molecule in its ground state (Equation (1)). After this, two radicals undergo disproportionation as shown in Equation (2)) [28].

[D^+^:I^–^] + I^–^ → D + I_2_ (1)

2I_2_˙^–^ → I^–^ + I_3_^–^ (2)

Dye regeneration therefore depends upon the presence of I^–^ ions in the electrolyte solution, and it has been established that an increase in I^–^ concentration can be beneficial for DSC performance [30]. Wang et al. have demonstrated that for solvent-free electrolytes with ionic liquids (ILs) as media, a high I^–^ concentration is essential for efficient dye regeneration [31]. On the other hand, a high concentration of I^–^ may lead to undesired quenching of the excited-state dye, D* (Equation (3)) [31]. Too high an I^–^ concentration can lead to the formation of ion-pairs which have reduced mobility. This effect is especially observed in gel electrolytes [32].

D* + 2I^–^ → D^–^ + I_2_^–^ (3)

The concentration of I_3_^–^ ions is of importance for well-performing DSCs and directly affects the value of *V*_OC_ due to changes in the potential of the redox couple [33]. The formation constant for triiodide (equilibrium 4) is usually high in organic solvents; in MeCN, log *K* = 6.76 at 298 K [34]. At the same time, the concentration of I^–^ ions in the electrolyte is typically much higher than that of I_2_ [28]. We have previously shown that an electrolyte composed of LiI (0.18 M), I_2_ (0.05 M), and the IL 1-propyl-2,3-dimethylimidazolium iodide (PDMII, Scheme 1, 0.60 M) in methoxypropionitrile (MPN) led to a photoconversion efficiency of up to 0.66% for fully masked DSCs [25]. At the time of publication [25], this was the highest observed overall efficiency for an iron(II)-NHC sensitizer. In the latter electrolyte, the total concentration of I^–^ is 0.78 M (LiI 0.18 M in combination with PDMII 0.60 M), while the starting concentration of I_2_ is only 0.05 M.

I_2_ + I^–^ ⇌ I_3_ (4)

**Scheme 2:**
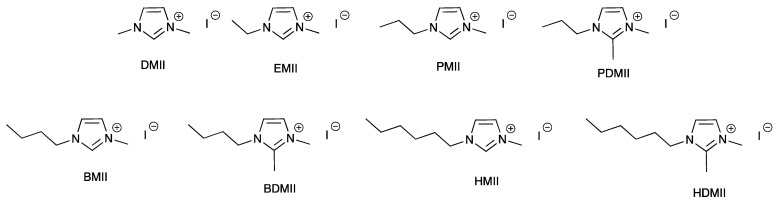
The structures of the ionic liquids (ILs) 1,3-dimethylimidazole iodide (DMII), 1-ethyl-3-methylimidazole iodide (EMII), 1-propyl-3-methylimidazole iodide (PMII), 1-propyl-2,3-dimethylimidazole iodide (PDMII), 1-butyl-3-methylimidazole iodide (BMII), 1-butyl-2,3-dimethylimidazole iodide (BDMII), 1-hexyl-3-methylimidazole iodide (HMII), and 1-hexyl-2,3-dimethylimidazole iodide (HDMII).

Using the ruthenium(II) dye N719, it has been demonstrated that an increase in I_2_ concentration has a direct influence on *J*_SC_ [35]. The optimal I_2_ concentration for the electrolyte in acetonitrile was 0.03 M. The remaining electrolyte components were 1-propyl-3-methylimidazolium iodide (PMII, Scheme 2, 1.00 M), guanidinium thiocyanate (0.10 M), and 1-methylbenzimidazole (MBI, 0.50 M). Upon going from lower to higher I_2_ concentrations (0.03 M to 0.20 M to 0.50 M), a positive shift of the redox potential was observed. Despite this shift, the *V*_OC_ values were not affected by different I_2_ concentrations. Interestingly, the use of the IL 1-ethyl-3-methylimidazolium tetracyanoborate (EMIBCN) as the solvent led to a different trend and 0.20 M I_2_ was the most beneficial concentration. It was also shown that the effective diffusion coefficient for I_3_^–^ was in a good agreement with the literature for the IL electrolyte, while for MeCN media, it was one order of magnitude lower than expected [35].

Significant differences between acetonitrile and EMIBCN lie in their density and viscosity, with the latter having a noteworthy influence on ion diffusion in the electrolyte. Table 1 gives the densities and viscosities of MeCN, EMIBCN, and MPN. The I_3_^–^ diffusion capability directly contributes to the DSC efficiency. Since the viscosity of electrolyte directly influences the transport of the components of the redox shuttle, more viscous solvents retard ion diffusion, and higher [I_3_]^–^ concentrations can help to overcome this limitation [35]. At the same time, a greater number of recombination processes involving the redox shuttle can occur. Hence, it is essential that the I_2_ concentration is optimized to provide the best possible electrolyte composition to enhance DSC performance.

In previous investigations, we have shown that the structure of the IL strongly influences the photoconversion efficiency of DSCs sensitized with the iron(II) dye **1** [25]. Here, we complement these results with a study of the effects on DSC performance of different I_2_ concentrations in the presence of different ILs with MPN as the electrolyte solvent. The dye throughout the investigation is **1** [23] (Scheme 2).

## 2. Materials and Methods

### 2.1. DSC Fabrication

Commercial FTO/TiO_2_ electrodes (Solaronix Test Cell Titania Electrodes, Solaronix SA, Aubonne, Switzerland) were rinsed with water, EtOH and dried on a heating plate at 450 °C for 30 min. Afterwards, the electrodes were cooled to 60 °C and immersed in the dye bath. For **1**, the dye bath consisted of **1** (0.50 mM) and chenodeoxycholic acid (cheno, 0.10 mM) in MeCN and the dipping time was ca. 15 h. For the reference dye N719 (Solaronix SA, Aubonne, Switzerland), the dye bath was a solution of N719 (0.30 mM) in EtOH and the dipping time was ca. 15 h. The electrodes were removed from the dye baths and were rinsed with the solvent used in the dye bath and dried under a flow of N_2_. Counter electrodes (Solaronix Test Cell Platinum Electrodes, Solaronix SA, Aubonne, Switzerland) were rinsed with EtOH and then heated at 450 °C for 30 min to remove volatile organic impurities.

The working and counter electrodes were combined with a thermoplast hot-melt sealing foil (Solaronix Test Cell Gaskets, 60 µm, Solaronix SA, Aubonne, Switzerland) by pressing them together while heating. Afterwards, the electrolyte was introduced into the space between the electrodes through a pre-drilled hole in the counter electrode by vacuum backfilling. The composition of the electrolytes varied as described in Section 3. The hole was sealed with hot-melt sealing foil and a cover glass (Solaronix Test Cell Sealings and Solaronix Test Cell Caps, Solaronix SA, Aubonne, Switzerland). Finally, silver paint (SPI Supplies, West Chester, PA 19381-0656, USA) was applied on the edges of each electrode from the FTO side.

### 2.2. DSC, External Quantum Efficiency (EQE) and Electrochemical Impedance Spectroscopy (EIS) Measurements

Current density-voltage (*J–V*) measurements were made by irradiating from the photoanode side with a LOT Quantum Design LS0811 instrument (LOT-QuantumDesign GmbH, Darmstadt, Germany, 100 mW cm^–2^ = 1 sun at AM 1.5) and the simulated light power was calibrated with a silicon reference cell. 

For the EIS measurements, a ModuLab® XM PhotoEchem photoelectrochemical measurement system (Solartron Metrology Ltd., Leicester, UK) was used. The impedance was measured in galvanostatic mode at the open-circuit potential of the cell at a light intensity of 22 mW cm^–2^ (590 nm) in the frequency range 0.05 Hz to 100 kHz with an amplitude of 10 mV. The impedance data were analyzed and fitted using ZView® sofware (Scribner Associates Inc., Southern Pines, NC, USA).

## 3. Results and discussion

### 3.1. Comparison of Electrolytes Containing 0.05 M and 0.10 M I_2_

In the first part of the investigation, DSCs sensitized with **1** in the presence of the co-adsorbant cheno were fabricated with electrolytes that differed in the ionic liquid present. The ILs shown in Scheme 1 were chosen as ionic liquid components, and the general composition was LiI (0.18 M), I_2_ (0.10 or 0.05 M), IL (0.60 M) in MPN. An additional electrolyte, PDMII_a, was made which contained the additive MBI; its composition was LiI (0.18 M), I_2_ (0.10 M), MBI (0.50 M), and IL (0.60 M) in MPN. We recently published statistical data to evaluate the reproducibility of DSCs, and demonstrated the need to measure data for multiple devices, and the legitimacy of using average values for the parameters [39]. In the following discussion, the average values of *J*_SC_, *V*_OC_, fill-factor (*ff*), and *η* are considered for appropriate comparison of DSCs sets between each other. DSC parameters extracted from *J–V* curves for all DSCs are given in Supplementary Table S1, and average values are presented in Table 2. The ruthenium(II) dye N719 was used as a reference. 

Firstly, we consider the 1-alkyl-3-methylimidazolium iodide-based electrolytes. Values of *J*_SC_ are remarkably similar for DSCs containing the electrolytes DMII and EMII with 3.82 and 3.86 mA cm^–2^, respectively (Table 2). Then, a decrease in *J*_SC_ is observed for the electrolytes with PMII (2.48 mA cm^–2^) and BMII (2.42 mA cm^–2^), and a comparable *J*_SC_ value of 2.60 mA cm^–2^ is observed for the DSCs with electrolyte HMII. Values of *V*_OC_ lie in the same range (297–269 mV) for DSCs with electrolytes DMII, PMII, and BMII, but are considerably lower for EMII and HMII (201 and 146 mV, respectively). Significantly, the *ff* values for all DSCs containing EMII and HMII are low (Supplementary Table S1) and low average values of *ff* in Table 1 are appropriately representative. The data for the devices with 1-alkyl-3-methylimidazolium iodide-based electrolytes reveal that the highest average *η* value of 0.65% corresponds to the IL with the shortest alkyl chain (DMII). We note that all four DSCs with this IL perform well, with values of *η* in the range 0.62–0.67%, which represents 10.0–10.8% of the photoconversion efficiency observed for DSCs sensitized by N719 (Supplementary Table S1).

Upon going from ILs with a 1-alkyl-3-methylimidazolium cation to ILs containing 1-alkyl-2,3-dimethylimidazolium cations (Scheme 1), noticeable changes in performance are observed. For example, DSCs with PDMII as the IL have higher *J*_SC_ values than those with PMII (Table 2 and Supplementary Table S1). The average value increases from 2.48 to 3.50 mA cm^–2^. At the same time, the decrease in *V*_OC_ and *ff* resulted in lower overall photoconversion efficiencies. A comparison of DSCs with electrolytes BDMII or BMII reveals no significant changes, and both average *η* values are 0.42% (Table 2). In the case of HDMII, the trends in parameters are different. While *J*_SC_ remains approximately constant on going from HMII to HDMII, values of *V*_OC_ and *ff* increase, and this leads to enhanced values of *η* (Table 2 and Supplementary Table S1).

**Figure 2:**
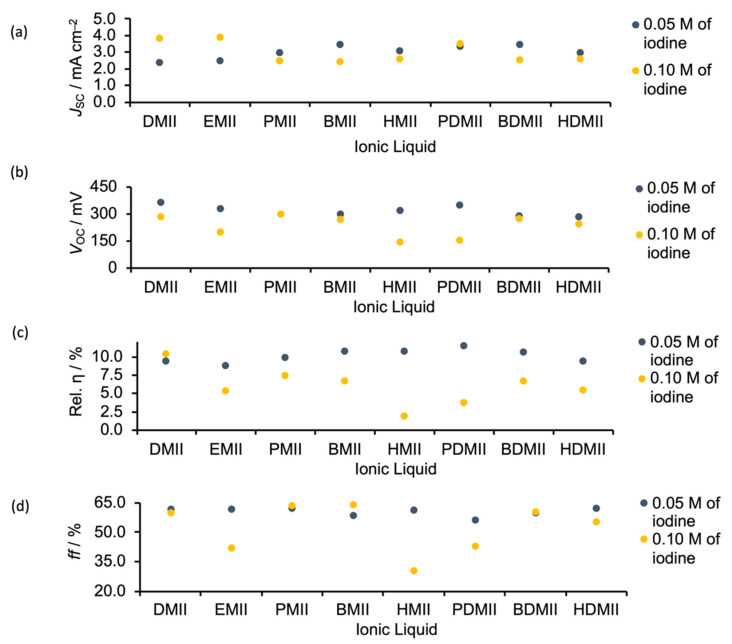
Average (**a**) *J*_SC_, (**b**) *V*_OC_ and (**c**) *η*_relative_ values (*η*_relative_ is with respect to N719) and (**d**) *ff* values for DSCs based on electrolytes with 0.05 or 0.10 M I_2_ and 1-alkyl-3-methylimidazolium or 1-alkyl-2,3-dimethylimidazolium iodide to illustrate the influence of iodine concentration and IL structure. Average values for 0.10 M I_2_ and for BDMII and HDMII ILs with 0.05 M I_2_ are from Table 2, and for other cells with 0.05 M I_2_, average values are calculated from the data in reference [25].

A comparison of the results from the current investigation with those reported earlier [25] are summarized in Figure 2. DSCs with electrolytes based on BDMII and HDMII ILs in the presence of 0.05 M I_2_ were not previously published, and their performance parameters are shown in Table 2 and Supplementary Table S1 (electrolytes BDMII_a and HDMII_a). At an I_2_ concentration of 0.05 M, the optimal IL was PDMII [25], but with 0.10 M I_2_, DMII gave the highest DSC performance. Cells with PDMII suffered from low values of *V*_OC_ and *ff* (Table 2 and Figure 2). Since values of *J*_SC_ were promising (average 3.50 mA cm^–2^, Table 2), it was decided to add MBI to the electrolyte since it has been established that MBI can improve *V*_OC_ [40,41]. However, although an increase in *V*_OC_ from 156 to 282 mV was observed (Table 2), it was accompanied by a dramatic reduction in *J*_SC_, and correspondingly low values of *η*.

The open circuit voltage is the difference between the Fermi level of a semiconductor and the redox potential of electrolyte. From the Nernst equation, an increase in the concentration of I^–^ results in a negative shift in redox potential for the I^–^/I_3_^–^ couple [33], consequently decreasing *V*_OC_ [33], and higher I_2_ concentrations lead to increased *V*_OC_. However, we observed (Figure 2b) that an increase in the concentration of I_2_ from 0.05 M to 0.10 M resulted in lower values of *V*_OC_ for all ILs. This suggests that, in these DSCs, the effect of the increase in I_2_ concentration on *V*_OC_ values has more to do with recombination processes [28] than the change in the redox potential of the I^–^/I_3_^–^ shuttle.

Considering *J*_SC_, higher values were obtained with an increased concentration of I_2_ only for DSCs containing DMII and EMII ILs. With PDMII, there was almost no change in *J*_SC_ (Figure 2) at different I_2_ concentrations. In the case of DSCs containing HMII or HDMII, the change in *J*_SC_ is less than 0.5 mA cm^–2^ (Figure 2a). The trend in *η* (irrespective of the IL with the exception of DMII) is for a lower electrolyte concentration of I_2_ to lead to higher performances (Figure 2c). 

### 3.2. EIS Measurements

In order to rationalize the observations discussed in Section 3.1, we performed EIS measurements on the DSCs. Fitted parameters for multiple DSCs are presented in Supplementary Table S2, and average values [39] are given in Table 3. Only the average values will be used for data comparison in the following discussion. For fitting experimental EIS data, the electric circuit model shown in Supplementary Figure S1 was used. The model includes transmission line impedance and Nernst diffusion impedance represented by a Warburg impedance. EIS data give insight into interfacial electronic processes. The electron lifetime (*τ)*, transport time (*τ*_t_), and diffusion length (*L*_d_) indicate the efficiency of electron collection on the back-side of the photoanode and contribute to the values of *J*_SC_. Diffusion resistance (*R*_d_) in the electrolyte is correlated to the mass transport of the redox species. 

**Figure 3:**
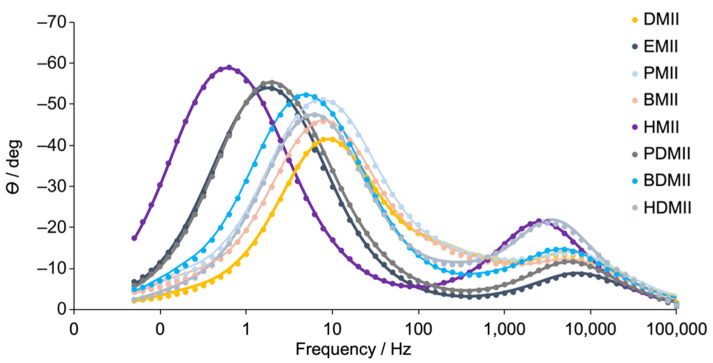
Bode plots of the most representative cells from each set of DSCs containing 0.10 M I_2_ in the electrolyte. Solid lines represent fitted curves, and circles represent experimental data.

As expected, the series resistance, *R*_S_, stays constant for all the DSCs. The resistance (*R*_Pt_) and capacitance (*C*_Pt_) of the counter electrodes are also invariant, except for cells which have HMII and HDMII ILs. These DSCs have the highest *R*_Pt_ values (12 and 9 Ω, respectively), and this can also be seen in the Bode plots (Figure 3, high frequency region). The differences in *R*_Pt_ may be a result of different interfacial contact between the platinum layer and the electrolyte. The variation in *ff* values (Table 2) may have a similar origin [42]. The process of catalytic I_3_^–^ reduction at the counter electrode is associated with a voltage loss due to overpotential, and this can cause a decrease in the fill-factor [43].

**Figure 4:**
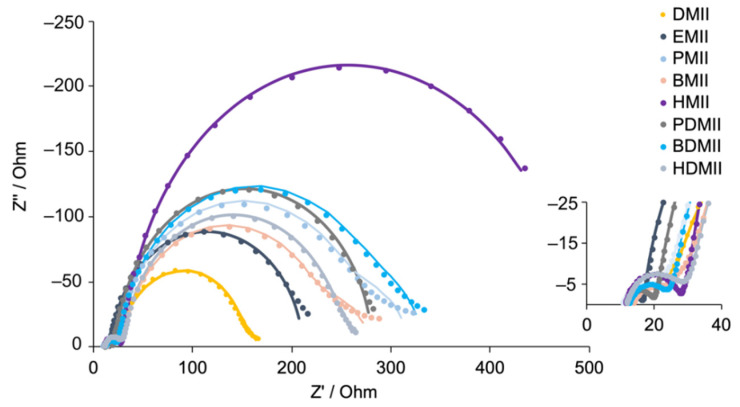
Nyquist plots for the most representative cells from each set of DSCs with 0.10 M I_2_. An expansion of high frequency region is shown in the inset. Solid lines represent fitted curves, and circle represent experimental data.

The transport of the components of the redox shuttle through the electrolyte medium is diffusion-driven and of key importance for efficient DSC performance [44]. High I^–^ concentrations are required for rapid dye regeneration. As the I^–^ concentration is always significantly greater than I_3_^–^, the I_3_^–^ concentration becomes rate limiting for mass transport in DSCs [35,43]. However, and in contrast to this, an excess of I_3_^–^ may lead to an increase in recombination processes at the photoanode. In addition to these effects, the I_3_^–^ ion has an absorption maximum around 360–380 nm in MPN [45], and leads to increased light absorption, but no electron injection [43]. In the Nyquist plots in Figure 4, the electrolyte contribution is observed in the low frequency region, and the counter electrode contribution in the high frequency region (expansion inset in Figure 4). The diffusion resistance *R*_d_ in the electrolyte allows us to probe the electron transfer at the counter electrode and should be considered together with the charge transfer resistance *R*_Pt_. The overall trend can be described as longer alkyl chains introduced into the IL correspond to higher *R*_d_ values (15 Ω for DMII compared to 80 Ω for HMII, Table 3). The exception is BMII, the average *R*_d_ value for which falls between those of EMII and PMII. The highest diffusion resistance corresponds to the highest *R*_Pt_ value for the HMII electrolyte (Table 3). For ILs with an additional methyl group (PDMII, BDMII, and HDMII), the average *R*_d_ values are in the range of 20–49 Ω (Table 3), with the highest diffusion resistance corresponding to the BDMII electrolyte. Interestingly, this did not affect *R*_Pt_, the value of which is comparable to other sets of DSC.

Another important parameter, which varies between cells, is the electron lifetime, *τ*. The longest *τ* values are observed for DSCs containing HMII in the electrolyte, and the shortest for the DMII electrolyte (Table 3 and Supplementary Table S2). The tendency for changes in *τ* is illustrated in the low frequency region of the Bode plots in Figure 3, since *τ* is inversely related to the maximum frequency [43,46]. The trends in electron lifetime are in agreement with the transport time, *τ*_t_, which must be lower than *τ* in order to ensure that there is effective transport of electrons through the TiO_2_ semiconductor [44]. The diffusion length, *L*_d_, is another crucial parameter for efficient electron transport in the semiconductor and should be longer than the active layer thickness [47]. For all electrolytes, *L*_d_ is longer than the TiO_2_ layer thickness (≈12 µm) and follows the trend in *τ* for all electrolytes. 

A trend similar to that for *τ* is observed for both the chemical capacitance, *C*_µ_, and the recombination resistance, *R*_rec_. These trends are as expected, because *τ* is directly related to *C*_µ_ and *R*_rec_ [43]. The increase in *R*_rec_ (Table 2) upon going from DMII (113 Ω) to HMII (397 Ω) is observed in both the Bode and Nyquist plots (Figure 3 and Figure 4). The variation in the average values of *R*_rec_ for the other ILs is not significant [39] and has no meaningful impact on the performance of the DSCs (Table 2).

The values of the chemical capacitance, *C*_µ_, are comparable for all DSCs, except for devices containing EMII, HMII and PDMII (Table 3 and Supplementary Table S2). Use of EMII and PDMII leads to remarkably high *J*_SC_ values (~3.50 mA cm^–2^), but all three sets have rather low values of *V*_OC_ (146–201 mV) in agreement with the chemical capacitance data. Overall, this results in low photoconversion. Interestingly, these cells also have low values of transport resistance *R*_tr_. For the other electrolytes, *R*_tr_ is in the range of 22–27 Ω except for BDMII, which has an average value of 9 Ω.

The increase in I_2_ concentration in the electrolyte from 0.05 M to 0.10 M resulted in different impedance responses as seen by comparing the entries in Table 3 and Table 4. While *R*_Pt_ and *C*_Pt_ stay constant, the diffusion resistance showed significant differences for all ILs as the I_2_ concentration increased. The decrease in *R*_rec_ for DMII and EMII brought a beneficial impact to *J*_SC_ (Figure 2). For the EMII electrolyte, the large increase in chemical capacitance as well as a decrease in *R*_tr_ are responsible for the higher *J*_SC_. Unfortunately, the beneficial changes in *C*_µ_ typically resulted in a decrease in *V*_OC_. In the case of electrolytes with PMII and BMII, an increase in the I_2_ concentration resulted in increased values of *R*_rec_, and therefore lower *J*_SC_ (Figure 2). For the BMII electrolyte, a slight increase in *ff* is observed. However, the increase in chemical capacitance for HMII from 311 µF (Table 4) to 2678 µF (Table 3) is followed by an increase in *R*_rec_. The values of *ff* decreased by a factor of two [25]. At the same time, a significant increase in *R*_d_ from 18 to 80 Ω is seen.

Overall, the increase in I_2_ concentration led to noteworthy changes, not only in the *J-V* plots for all ILs (see Supplementary Figures S2 and S3), but also in the impedance. The most beneficial changes were observed for DMII. Interesting trends were seen for electrolytes containing EMII, HMII, and PDMII ILs with a radical increase in chemical capacitance values. Moreover, it was noticed that the structure of the IL had an impact on the electrolyte/counter electrode interface, what results in different trends in *R*_Pt_.

### 3.3. Further Iodine Concentration Investigations for Electrolytes With DMII, EMII and PDMII ILs

Based on the results described above, three ILs (DMII, EMII and PDMII) were selected for an in-depth investigation of the effects of different I_2_ concentrations. As well as 0.02 and 0.20 M I_2_, we included electrolytes in which no I_2_ was added to the initial electrolyte. These electrolytes were colorless when they were introduced to the DSC. Each electrolyte in Table 5 contains an excess of I^–^ from a combination of the imidazolium iodide salts and LiI. Regeneration of the dye from its oxidized form by reaction with I^–^ converts I^–^ to I_3_^–^ (Equations (1) and (2) in the Introduction), and therefore the DSC is expected to operate without explicit addition of I_2_ [48]. Sets of multiple DSCs were fabricated with electrolytes having the compositions shown in Table 5. The parameters for fully-masked DSCs are given in Supplementary Table S3, and average values are presented in Table 6.

**Figure 5:**
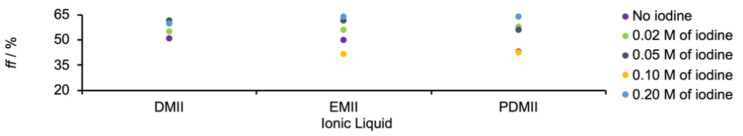
Values of the average fill factor values for fully-masked DSCs with different ILs in the electrolytes and different concentrations of I_2_.

Irrespective of the IL, the same trend in *ff* is observed upon going from electrolytes without I_2_ to 0.02 M and to 0.20 M I_2_. For electrolytes without iodine, the lowest fill factor values were measured. Higher I_2_ concentration led to an increase in *ff* values. The most significant changes occurred in the PDMII systems. An overall picture is gained from inspection of Figure 5 which combines data from Table 2 and Table 6, and average values calculated from data in reference [25] for 0.05 M I_2_.

**Figure 6:**
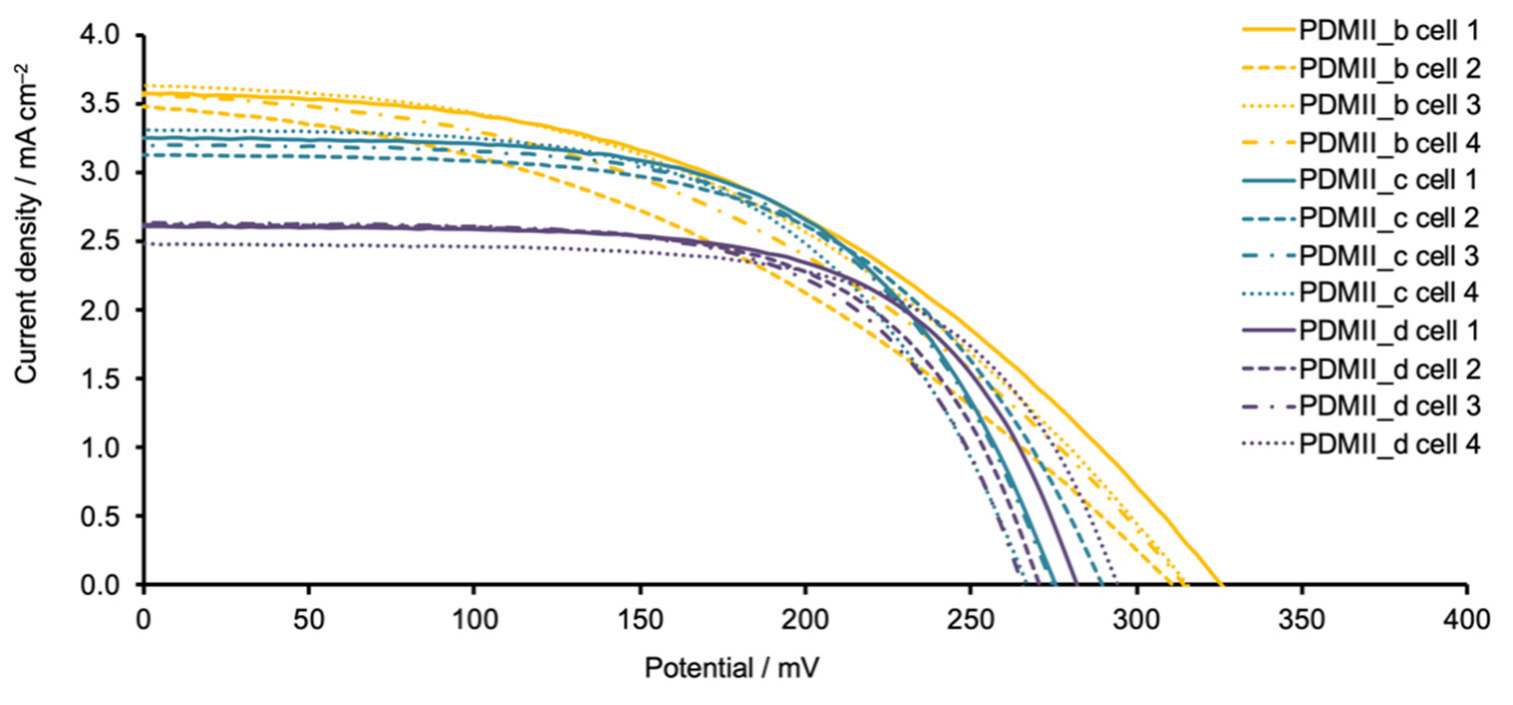
*J-V* curves for multiple, masked DSCs with electrolytes PDMII_b (no added I_2_), PDMII_c (0.02 M I_2_) and PDMII_d (0.20 M I_2_).

Supplementary Figure S4 summarizes the variation in average values of *J*_SC_, *V*_OC_ and photoconversion efficiencies relative to the reference DSCs with N719, for I_2_ concentrations from 0 to 0.20 M (Table 2 and Table 6, and averages calculated from our previous study [25]). The trends are not clear cut, but we conclude that too high an I_2_ concentration (0.20 M) is detrimental to performance, while 0.10 M I_2_ leads to high *J*_SC_, irrespective of IL, but to low *V*_OC_ when the IL is EMII or PDMII. The greatest efficiency difference is observed for 0.05 and 0.10 M I_2_ in PDMII based electrolytes with *η* = 0.65% (11.6 % relative to N719) and *η* = 0.25% (3.8% relative to N719), respectively. Figure 6 illustrates the *J*–*V* curves for all cells with the PDMII_b, PDMII_c, and PDMII_d electrolytes, demonstrating the reproducibility of the trends in performance.

### 3.4. EIS Measurements of DSCs With 0.20 M, 0.02 M and No I_2_ in the Electrolytes

EIS measurements were conducted for DSCs in which the electrolytes contained the ILs DMII, EMII, or PDMII with either no added I_2_, or 0.02, or 0.20 M I_2_ (for complete electrolyte compositions, see Table 5). The impedance was measured for multiple DSCs and the fitted parameters are given in Supplementary Table S4. Average values are given in Table 7 and these will be used in the following discussion.

**Figure 7:**
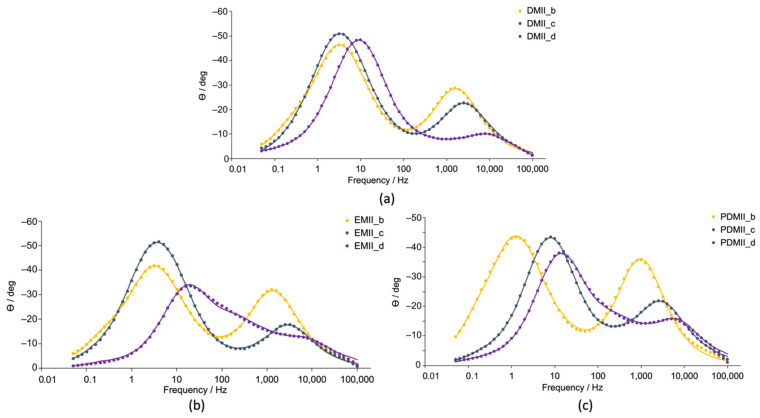
EIS Bode plots for DSCs with (**a**) electrolytes with DMII IL, (**b**) electrolytes with EMII, and (**c**) electrolytes with PDMII. Solid lines represent fitted curves, and circle represent experimental data. The yellow colour corresponds to electrolytes with no added I_2_, dark blue to electrolytes with 0.02 M I_2_, and purple to 0.20 M I_2_.

The series resistance, *R*_S_, remains constant for all the DSCs, as do values of *C*_Pt_ for the platinum counter electrode. Independent of the structure of the IL, the DSCs without iodine have the greatest *R*_Pt_ and *R*_d_ values that increase upon going from DMII to EMII to PDMII ILs. This can be illustrated with the Nyquist plots as the changes along the real (*Z*’) axis indicate the changes in the diffusion resistance (Supplementary Figures S5–S7). With the addition of 0.02 M I_2_, a decrease is observed for both parameters. A further increase of I_2_ concentration to 0.20 M is followed by a decrease in the values of *R*_Pt_ and *R*_d_. The trend in resistance at the counter electrode is inversely proportional to the trend in the fill factor, and the lowest values of *ff* (Table 6) correspond to electrolytes without added I_2_. The variation in *R*_Pt_ can be illustrated with the help of a Bode plot (Figure 7). The shift of the right-hand peak in each of Figure 7a–c from lower to higher frequencies upon going from 0.00 to 0.20 M I_2_ is attributed to a decrease in the charge transfer time [49], and is observed for each of the ILs. The structure of the IL also influences the EIS response for the DSCs. Thus, DSCs with an electrolyte with no added I_2_ and with methyl substituents in the IL (DMII) has a lower *R*_d_ than cells containing the electrolyte EMII_b (120 and 138 Ω, respectively, Table 7). Increasing the length of the substituent chain from EMII_b to PDMII_b increases *R*_d_ further to 256 Ω. On the other hand, DSCs with the electrolytes PDMII_d and EMII_d (0.20 M I_2_) show lower *R*_d_ values of 10 Ω compared to 24 Ω for DMII_d (Table 7).

**Figure 8:**
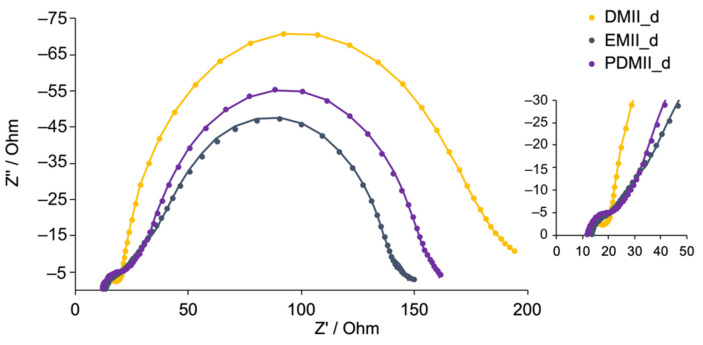
The Nyquist plot with high frequency region expansion of the DSCs with DMII_d, EMII_d and PDMII_d electrolytes to demonstrate the change in spectra profile depending on IL structure.

The diffusion length *L*_d_ was also affected by changes in the electrolyte compositions. In the case of the DMII family, DSCs containing electrolytes without added I_2_ or with 0.02 M I_2_ (DMII_b and DMII_c in Table 7) have a higher *L*_d_, at least by a factor of six, than the active layer thickness *d* (*d* ≈ 12 µm). For DMII_d, a significant decrease was observed, but still, the diffusion length is almost four times longer than the thickness of the TiO_2_ layer. In the case of cells containing EMII, the highest diffusion lengths are again observed for electrolyte with no added I_2_ or 0.02 M I_2_ (Table 7). Increasing the I_2_ concentration to 0.2 M resulted in a short *L*_d_ of 17 µm, which is comparable to the semiconductor thickness. This trend is also seen for DSCs in which the electrolyte contained the IL PDMII (Table 7). However, when *L*_d_ becomes shorter than the active layer thickness, the so-called Gerischer impedance (*Z*_G_) overtakes the diffusion-recombination impedance model [47]. Typically, when a large recombination rate is observed, the diffusion–recombination impedance needs to be changed to the Gerischer impedance [50]. In this case, the transport resistance *R*_tr_ becomes significantly larger than the recombination resistance *R*_rec_ and the recombination time is shorter than the diffusion time through the active layer [50,51]. Practically, *R*_rec_, *R*_tr_, and *C*_µ_ parameters cannot be extracted in the presence of *Z*_G_ (*L*_d_ << *d*) and *L*_d_ of ≈ 0.5*d* is the limit to obtain them. Thus, the diffusion-recombination impedance model used in this study is suitable for EIS fitting despite comparable values of *L*_d_ and *d* for EMII_d and PDMII_d electrolytes. At the same time, the shapes of the experimental curves for EMII_d and PDMII_d also change in the *Z*_G_ direction (Figure 8 and Figure 7b,c). The effect is particularly seen for the EMII_d electrolyte (Figure 7b, EMII_d).

For all DSC sets, the electron lifetime significantly decreased from the electrolyte with no added I_2_ to 0.20 M I_2_. This trend can be seen in the Bode plots with the shift of the left-hand peaks to higher frequencies (Figure 7) and this is particularly noticeable for the DSCs containing PDMII (Figure 7c). Nevertheless, *τ* is considerably larger than *τ*_t_ for all electrolytes. The transport resistance, *R*_tr_, increases for all electrolyte families upon going from no added I_2_ to 0.20 M I_2_. Interestingly, the lowest *R*_tr_ for electrolytes containing 0.20 M I_2_ is observed for DMII (12 Ω, Table 7), indicating an efficient transport compared to that in ILs EMII and PDMII (60 Ω, Table 7).

The lowest values of chemical capacitance as well as recombination resistance are observed for all electrolytes when the I_2_ concentration is 0.20 M (Table 7). The remarkably high *C*_µ_ for PDMII_b is consistent with the observed high values of *J*_SC_ (Table 6 and Supplementary Table S4), The dramatic fall in *C*_µ_ upon going from electrolyte PDMII_c to PDMII_d (Table 7) has a response in lower *J*_SC_ for this electrolyte. The recombination resistance follows the *C*_µ_ trend and supports the observed changes in values of *J*_SC_. From EMII_b to EMII_c, the fluctuation in *C*_µ_ is not substantial, but in combination with an increase in *R*_rec_ results to lower *J*_SC_. Upon going to EMII_d with 0.20 M I_2_, *R*_rec_ and *C*_µ_ both decrease and lead to a consistent drop in *J*_SC_. In the case of the DMII series, a drop in both *R*_rec_ and *C*_µ_ is observed as the concentration of I_2_ is increased and this is consistent with a decrease in *J*_SC_ values (Table 7 and Table 6, respectively). 

Overall, changes in the I_2_ concentration significantly influence most of the electronic processes in the DSCs, irrespective of the ionic liquid. The main trends were observed for resistance in the electrolyte, counter electrode resistance, transport resistance, and electron diffusion length in the semiconductor. 

## 4. Conclusions

We have investigated the effect of changing the I_2_ concentration in the electrolyte in DSCs sensitized with the Fe(II)-NHC dye **1** and containing the I^–^/I_3_^–^ redox shuttle and different ILs. Either no I_2_ was added to the initial electrolyte, or the initial concentrations were 0.02, 0.10, and 0.20 M. The study complements our earlier investigations where the electrolyte composition was LiI (0.18 M), I_2_ (0.05 M), and PDMII (0.60 M) in MPN as solvent [25]. In the operating DSC, the IL has an influence on the TiO_2_/electrolyte and electrolyte/counter-electrode interfaces. According to the EIS and *J-V* measurements, it was shown that the structure of the IL influences various parameters including the diffusion resistance in the electrolyte, resistance at the counter electrode and the charge diffusion in the semiconductor. Values of *J*_SC_, *V*_OC_, and the fill factor were influenced by changes in the I_2_ concentration for all ILs used in this study. The most dramatic changes were observed when the IL was HMII, and the poor performances of these DSCs arose from low *V*_OC_ and low fill factors. The limiting processes correlated to increased electrolyte diffusion resistance and charge transfer resistance at the platinum counter electrode. Interestingly, DSCs containing the ILs with the shortest alkyl-chains (DMII and EMII) showed an increase in *J*_SC_ upon going from 0.05 to 0.10 M I_2_ compared to ILs with longer side chains. 

In the case of the ILs DMII, EMII, and PDMII, it was shown that cells in which there was no added I_2_ or a concentration of I_2_ lower than 0.05 M I_2_ exhibited the lowest values of the transport resistance in the photoanode semiconductor. At the same time, higher I_2_ concentrations led to a decrease in diffusion resistance in the electrolyte as well as in the platinum counter electrode resistance. The electron lifetime and diffusion length were also affected by the I_2_ concentration, and the values decreased upon going from no added I_2_ to 0.20 M I_2_. A similar trend is observed in *J*_SC_ for DMII, EMII and PDMII ILs. Considering all aspects in this study, DMII was the best performing IL, not only in terms of *η*, but it also had the most promising impedance profile. A combination of DMII and 0.1 M I_2_ in the electrolyte produced the best performing DSCs with *η* values in the range 0.62–0.67%, which correspond to 10.0–10.8% of the photoconversion efficiency observed for DSCs sensitized by N719. Despite the high values of *J*_SC_ and *V*_OC_ in DSCs with DMII where no I_2_ was added to the initial electrolyte, these cells, as anticipated, suffered from low *ff* values, and consequently low *η*.

## Figures and Tables

**Table 1:** Solvent densities and viscosities at room temperature [36,37,38].

Solvent	Density at 298 K /g mL^–1^	Viscosity /mPa s ^1^
Acetonitrile	0.786	0.37 (298 K)
1-Ethyl-3-methylimidazolium tetracyanoborate (EMIBCN)	1.294	19.8 (293 K)
3-Methoxypropionitrile (MPN)	0.937	2.5 (298 K)

^1^ 1 cP = 1 mPa s.

**Table 2:** Average values of DSC parameters for sets of multiple DSCs using electrolytes with different ionic liquids. The electrolyte composition for DMII, EMII, PMII, BMII, PDMII and BDMII was LiI (0.18 M), I_2_ (0.10 M), IL (0.60 M) in MPN; for PDMII_a, the composition was LiI (0.18 M), I_2_ (0.10 M), PDMII (0.60 M), MBI (0.50 M) in MPN; for BDMII_a and HDMII_a, the composition was LiI (0.18 M), I_2_ (0.05 M), PDMII (0.60 M) in MPN. Electrolytes are labelled according to the IL present.

Electrolyte Name	*J*_SC_/mA cm^–2^	*V*_OC_/mV	*ff*/%	*η*/%	Rel. *η*/% ^1^
DMII	3.82	285	60	0.65	10.5
EMII	3.86	201	42	0.34	5.5
PMII	2.48	297	63	0.47	7.6
BMII	2.42	269	64	0.42	6.7
HMII	2.60	146	31	0.12	1.9
PDMII	3.50	156	43	0.24	3.8
BDMII	2.55	272	60	0.42	6.8
HDMII	2.58	242	55	0.35	5.6
PDMII_a	0.07	282	51	0.01	0.2
BDMII_a	3.48	290	60	0.60	10.7
HDMII_a	2.99	285	62	0.53	9.4

^1^ Relative an N719-sensitized DSC efficiency of 6.19% (average of three cells with *η* = 6.22, 6.21 and 6.14%).

**Table 3:** Average EIS parameters ^1^ for DSCs with 0.10 M I_2_ in electrolytes. See Table S2 for all data.

Electrolyte Name	*R*_rec_/Ω	*C*_µ_/µF	*R*_tr_/Ω	*τ*/ms	*τ*_t_/ms	*L*_d_/µm	*R*_d_/Ω	*R*_S_/Ω	*R*_Pt_/Ω	*C*_Pt_/µF
DMII	113	413	26	47	10	26	15	10	4	6
EMII	202	1531	2	319	3	137	28	11	6	6
PMII	229	350	22	80	8	39	60	11	4	6
BMII	186	350	27	66	8	36	47	13	6	5
HMII	397	2678	1	3991	3	240	80	11	12	6
PDMII	240	1166	2	282	3	124	20	12	6	5
BDMII	230	450	9	103	4	60	49	13	7	5
HDMII	240	352	25	85	9	37	26	12	9	6

^1^*R*_rec_ = recombination resistance; *C*_μ_ = chemical capacitance; *R*_tr_ = transport resistance; *R*_S_ = series resistance; *R*_Pt_ = resistance of the counter electrode; *C*_Pt_ = capacitance of the counter electrode; *R*_d_ = diffusion resistance; *L*_d_ = diffusion length; *τ*_t_ = transport time; *τ* = electron lifetime.

**Table 4:** EIS parameters for DSCs containing electrolytes with 0.05 M I_2_ ^1^.

Electrolyte Name	*R*_rec_/Ω	*C*_µ_/µF	*R*_tr_/Ω	*τ*/ms	*τ*_t_/ms	*L*_d_/µm	*R*_d_/Ω	*R*s/Ω	*R*_Pt_/Ω	*C*_Pt_/µF
DMII	313	508	16	164	7	67	43	12	5	7
EMII	315	468	16	148	8	55	36	12	7	5
PMII	136	323	18	44	5	36	10	13	9	6
BMII	152	358	12	55	4	46	10	14	9	5
HMII	169	311	40	53	12	26	18	13	11	5

^1^ Average values have been calculated from data in reference [25].

**Table 5:** Electrolyte compositions with different I_2_ concentrations and DMII, EMII or PDMII IL. The solvent was MPN in all electrolytes.

Electrolyte Name	Concentration of IL/M	LiI/M	I_2_/M
DMII_b	0.60	0.18	none added
DMII_c	0.60	0.18	0.02
DMII_d	0.60	0.18	0.20
EMII_b	0.60	0.18	none added
EMII_c	0.60	0.18	0.02
EMII_d	0.60	0.18	0.20
PDMII_b	0.60	0.18	none added
PDMII_c	0.60	0.18	0.02
PDMII_d	0.60	0.18	0.20

**Table 6:** Average values of the parameters for fully-masked DSCs using electrolytes with the compositions detailed in Table 5. See Table S3 in the Supporting Materials for all data.

Electrolyte Name	*J*_SC_/mA cm^–2^	*V*_OC_/mV	*ff*/%	*η*/%	Rel.*η* /% ^1^
DMII_b	3.13	297	51	0.47	7.4
DMII_c	3.17	300	55	0.52	8.2
DMII_d	2.76	278	60	0.46	7.2
EMII_b	3.32	286	50	0.48	7.4
EMII_c	3.10	290	56	0.50	7.9
EMII_d	2.56	272	64	0.45	7.0
PDMII_b	3.56	317	43	0.49	7.7
PDMII_c	3.22	277	58	0.52	8.1
PDMII_d	2.59	278	64	0.46	7.2

^1^ Relative to an N719-sensitized DSC efficiency of 6.41% (average of two cells each with *η* = 6.41%).

**Table 7:** Average values of the EIS parameters for DSCs with 0.00, 0.02 and 0.20 M I_2_ in the electrolytes. The electrolyte compositions are defined in Table 5.

Electrolyte Name	*R*_rec_/Ω	*C*_µ_/µF	*R*_tr_/Ω	*τ*/ms	*τ*_t_/ms	*L*_d_/µm	*R*_d_/Ω	*R*_S_/Ω	*R*_Pt_/Ω	*C*_Pt_/µF
DMII_b	243	517	7	127	3	72	120	13	24	7
DMII_c	269	626	7	168	5	83	40	12	19	6
DMII_d	149	357	12	53	4	43	24	11	4	6
EMII_b	192	697	10	133	7	55	138	12	33	8
EMII_c	237	618	7	147	4	73	32	13	12	6
EMII_d	109	189	60	21	11	16	10	12	3	8
PDMII_b	233	1294	11	300	14	57	256	13	44	8
PDMII_c	168	340	24	57	8	32	21	11	20	6
PDMII_d	121	193	62	23	12	17	10	11	5	7

## Data Availability

The data presented in this study are available on request from the corresponding author. The data are not publicly accessible at present.

## Supplementary Materials

The following are available online at https://www.mdpi.com/article/10.3390/ma14113053/s1, Table S1: Parameters for sets of multiple, fully-masked DSCs using electrolytes with different ionic liquids, and electrolyte composition of LiI (0.18 M), I2 (0.10 M), IL (0.60 M) in MPN; for PDMII_a: LiI (0.18 M), I2 (0.10 M), PDMII (0.60 M), MBI (0.50 M) in MPN; for BDMII_a and HDMII_a: LiI (0.18 M), I2 (0.05 M), PDMII (0.60 M) in MPN; Table S2: EIS parameters for all DSCs with 0.10 M I2 in the electrolytes; Table S3: Parameters for sets of multiple, fully-masked DSCs using electrolytes with the compositions detailed in Table 5; Table S4: EIS parameters for DSCs with 0.00, 0.02 and 0.20 M I_2_ in the electrolytes. The electrolyte compositions are defined in Table 5; Figure S1: The equivalent circuit model used in the EIS study. This includes a series resistance (*R*s), a resistance (*R*_Pt_) and a constant phase element (CPE1) to model a counter electrode, an extended distributed element (DX1) to represent the mesoporous TiO_2_/electrolyte interface as a transmission line model, and a Warburg element (Ws1), which represents the diffusion of the electrolyte; Figure S2: *J*–*V* curves for DSCs containing electrolytes with different ionic liquids (see Scheme 2 for abbreviations) and 0.05 M I_2_ in the initial electrolyte; Figure S3: *J*–*V* curves for DSCs containing electrolytes with different ionic liquids (see Scheme 2 for abbreviations) and 0.10 M I_2_ in the initial electrolyte; Figure S4: Average values of (**a**) *J*_SC_, (**b**) *V*_OC_ and (**c**) *η* (relative to N719) for masked DSCs with different ILs and I_2_ concentrations in the electrolytes; Figure S5: EIS Nyquist plots for DSCs with electrolytes with DMII IL. Solid lines represent fitted curves, and circle represent experimental data. The yellow colour corresponds to electrolytes with no added I_2_, dark blue to electrolytes with 0.02 M I_2_, and purple to 0.20 M I_2_; Figure S6: EIS Nyquist plots for DSCs with electrolytes with EMII IL. Solid lines represent fitted curves, and circle represent experimental data. The yellow colour corresponds to electrolytes with no added I_2_, dark blue to electrolytes with 0.02 M I_2_, and purple to 0.20 M I_2_; Figure S7: EIS Nyquist plots for DSCs with electrolytes with PDMII IL. Solid lines represent fitted curves, and circle represent experimental data. The yellow colour corresponds to electrolytes with no added I_2_, dark blue to electrolytes with 0.02 M I_2_, and purple to 0.20 M I_2_
