# Supplementary material for: Electrolyte Tuning in Iron(II)-Based Dye-Sensitized Solar Cells: Different Ionic Liquids and I2 Concentrations
Source: Materials (Basel). 2021 Jun 3;14(11):3053. doi: 10.3390/ma14113053 (PMC8200003; doi:10.3390/ma14113053)
Supplement: Supplementary file 1 [file materials-14-03053-s001.zip › materials-1244117-supplementary.pdf]

materials

MDPI

Supplementary Materials

Electrolyte Tuning in Iron(II)-Based Dye-Sensitized Solar Cells: Different Ionic Liquids and I_2 concentrations

Mariia Becker, Catherine E. Housecroft and Edwin C. Constable *

Department of Chemistry, University of Basel, BPR 1096, Mattenstrasse 24a, CH-4058 Basel, Switzerland; mariia.karpacheva@unibas.ch (M.B.); catherine.housecroft@unibas.ch (C.E.H.);

* Correspondence: edwin.constable@unibas.ch (Tel +41 61 207 1001)

Table S1. Parameters for sets of multiple, fully-masked DSCs using electrolytes with different ionic liquids, and electrolyte composition of LiI (0.18 M), I_2 (0.10 M), IL (0.60 M) in MPN; for PDMII_a: LiI (0.18 M), I_2 (0.10 M), PDMII (0.60 M), MBI (0.50 M) in MPN; for BDMII_a and HDMII_a: LiI (0.18 M), I_2 (0.05 M), PDMII (0.60 M) in MPN.

Entry	Electrolyte	J_{SC} / mA cm^{-2}	V_{OC} / mV	*ff* / %	η / %	Rel. η / % [1]
1	DMII cell 1	3.94	282	61	0.67	10.8
	DMII cell 2	3.70	285	60	0.64	10.3
	DMII cell 3	3.93	275	58	0.62	10.0
	DMII cell 4	3.70	296	61	0.67	10.8
2	EMII cell 1	3.50	211	41	0.30	4.8
	EMII cell 2	4.69	234	48	0.53	8.6
	EMII cell 3	2.86	173	38	0.19	3.1
	EMII cell 4	4.39	187	40	0.33	5.3
3	PMII cell 1	2.55	288	64	0.47	7.6
	PMII cell 2	2.37	297	64	0.45	7.3
	PMII cell 3	2.53	297	63	0.48	7.7
	PMII cell 4	2.48	306	63	0.47	7.7
4	BMII cell 1	2.45	274	64	0.43	6.9
	BMII cell 2	2.19	263	64	0.37	6.0
	BMII cell 3	2.56	275	64	0.45	7.3
	BMII cell 4	2.48	266	63	0.42	6.7
5	HMII cell 1	2.95	164	32	0.16	2.6
	HMII cell 2	2.84	166	32	0.15	2.4
	HMII cell 3	2.10	115	29	0.07	1.1
	HMII cell 4	2.52	138	29	0.10	1.6
6	PDMII cell 1	3.51	171	47	0.28	4.5
	PDMII cell 2	3.69	156	42	0.24	3.9
	PDMII cell 3	3.34	154	43	0.22	3.6
	PDMII cell 4	3.45	144	39	0.20	3.2
7	BDMII cell 1	2.56	283	61	0.44	7.2
	BDMII cell 2	2.59	262	60	0.40	6.5
	BDMII cell 3	2.59	282	60	0.44	7.1
	BDMII cell 4	2.45	260	61	0.39	6.2
8	HDMII cell 1	2.45	251	57	0.35	5.7
	HDMII cell 2	2.59	229	56	0.33	5.3

	HDMII cell 3	2.51	229	56	0.32	5.2
	HDMII cell 4	2.76	259	52	0.38	6.1
9	PDMII_a cell 1	0.07	285	51	0.01	0.2
	PDMII_a cell 2	0.07	278	50	0.01	0.2
10	BDMII_a cell 1	3.70	279	58	0.60	10.7
	BDMII_a cell 2	3.30	321	61	0.64	11.5
	BDMII_a cell 3	3.37	295	61	0.61	10.9
	BDMII_a cell 4	3.53	263	59	0.55	9.8
11	HDMII_a cell 1	3.00	303	63	0.57	10.2
	HDMII_a cell 2	3.04	254	60	0.46	8.2
	HDMII_a cell 3	3.04	282	62	0.53	9.5
	HDMII_a cell 4	2.87	302	63	0.55	9.8

[1] Relative to average N719 efficiency of 6.19% from all N719 values (6.22, 6.21 and 6.14%) during DSCs measurements.

Table S2. EIS parameters for all DSCs with 0.10 M I_2 in the electrolytes.

Entry	Electrolyte	R_{rec} / Ω	C_{μ} / μF	R_{tr} / Ω	τ / ms	τ_t / ms	L_d / μm	R_d / Ω	R_s / Ω	R_{Pt} / Ω	C_{Pt} / μF
1	DMII cell 1	86	428	20	37	8	25	10	9	5	6
	DMII cell 2	115	385	33	44	13	23	21	9	5	6
	DMII cell 3	127	477	16	60	8	34	15	9	4	6
	DMII cell 4	124	363	34	45	12	23	14	11	5	6
2	EMII cell 1	284	1796	2	510	4	133	22	11	8	6
	EMII cell 2	88	1256	2	111	3	76	37	11	7	5
	EMII cell 3	252	1306	1	329	1	191	44	11	4	6
	EMII cell 4	184	1764	1	325	2	147	10	11	5	6
3	PMII cell 1	188	355	19	67	7	38	42	12	5	6
	PMII cell 2	248	335	30	83	10	35	72	11	4	7
	PMII cell 3	248	383	17	95	6	46	65	11	4	6
	PMII cell 4	231	328	24	76	8	37	61	11	5	6
4	BMII cell 1	195	431	12	84	5	48	41	15	5	5
	BMII cell 2	192	309	32	59	10	29	55	14	5	5
	BMII cell 3	195	402	16	78	6	42	51	11	6	5
	BMII cell 4	164	259	49	42	13	22	41	12	6	6
5	HMII cell 1	382	2582	3	986	7	147	72	12	15	6
	HMII cell 2	374	2435	1	910	2	238	87	11	10	6
	HMII cell 3	457	2849	1	13000	2	344	83	11	8	7
	HMII cell 4	376	2845	1	1068	3	233	77	11	13	6
6	PDMII cell 1	182	954	2	174	2	115	33	13	6	5
	PDMII cell 2	241	1324	2	320	3	132	21	12	7	5
	PDMII cell 3	297	1136	3	338	3	127	15	13	7	5
	PDMII cell 4	239	1250	2	298	3	122	11	11	5	5
7	BDMII cell 1	244	407	13	99	5	52	48	12	6	5
	BDMII cell 2	249	459	9	114	4	64	57	13	9	5
	BDMII cell 3	196	485	7	95	3	63	43	13	6	5
8	HDMII cell 1	215	367	22	79	8	38	18	12	12	5
	HDMII cell 2	164	319	28	52	9	29	21	12	8	5
	HDMII cell 3	329	328	28	108	9	41	44	12	8	6
	HDMII cell 4	251	396	23	100	9	40	22	12	10	6

Table S3. Parameters for sets of multiple, fully-masked DSCs using electrolytes with the compositions detailed in Table 5.

Entry	Electrolyte	J_{SC} / mA cm^{-2}	V_{OC} / mV	*ff* / %	η / %	Rel. η / % [1]
1	DMII_b cell 1	3.02	316	50	0.48	7.5
	DMII_b cell 2	3.28	294	52	0.50	7.7
	DMII_b cell 3	3.06	285	52	0.46	7.1
	DMII_b cell 4	3.15	294	50	0.47	7.3
2	DMII_c cell 1	3.14	296	56	0.52	8.1
	DMII_c cell 2	3.25	298	55	0.53	8.3
	DMII_c cell 3	3.06	305	55	0.51	8.0
	DMII_c cell 4	3.23	300	55	0.53	8.3
3	DMII_d cell 1	2.51	291	63	0.46	7.2
	DMII_d cell 2	2.91	261	58	0.44	6.9
	DMII_d cell 3	2.84	282	60	0.48	7.5
	DMII_d cell 4	2.78	279	61	0.47	7.3
4	EMII_b cell 1	3.42	281	51	0.49	7.7
	EMII_b cell 2	3.22	294	52	0.49	7.7
	EMII_b cell 3	3.24	282	50	0.45	7.1
	EMII_b cell 4	3.42	286	48	0.47	7.3
5	EMII_c cell 1	3.18	293	56	0.52	8.1
	EMII_c cell 2	3.18	298	56	0.53	8.3
	EMII_c cell 3	3.22	286	56	0.51	8.0
	EMII_c cell 4	2.83	283	57	0.45	7.1
6	EMII_d cell 1	2.32	274	64	0.40	6.3
	EMII_d cell 2	2.41	272	63	0.42	6.5
	EMII_d cell 3	2.78	276	64	0.49	7.7
	EMII_d cell 4	2.74	267	64	0.47	7.3
7	PDMII_b cell 1	3.57	326	46	0.53	8.3
	PDMII_b cell 2	3.48	311	40	0.43	6.7
	PDMII_b cell 3	3.63	316	45	0.52	8.0
	PDMII_b cell 4	3.56	314	43	0.48	7.5
8	PDMII_c cell 1	3.25	276	59	0.53	8.3
	PDMII_c cell 2	3.12	290	58	0.52	8.1
	PDMII_c cell 3	3.20	275	60	0.52	8.2
	PDMII_c cell 4	3.31	267	57	0.51	7.9
9	PDMII_d cell 1	2.61	282	65	0.48	7.4
	PDMII_d cell 2	2.62	271	64	0.46	7.1
	PDMII_d cell 3	2.63	266	64	0.45	7.0
	PDMII_d cell 4	2.48	295	65	0.47	7.4

[1] Relative to average N719 efficiency of 6.19% from all N719 values (6.22, 6.21 and 6.14%) during DSCs measurements.

Table S4. EIS parameters for DSCs with 0.00, 0.02 and 0.20 M I_2 in the electrolytes. The electrolyte compositions are defined in Table 5.

Entry	Electrolyte	R_{rec} / Ω	C_{μ} / μF	R_{tr} / Ω	τ / ms	τ_t / ms	L_d / μm	R_d / Ω	R_S / Ω	R_{Pt} / Ω	C_{Pt} / μF
1	DMII_b cell 1	304	526	8	160	4	76	88	12	23	7
	DMII_b cell 2	230	608	6	140	4	73	133	12	23	7
	DMII_b cell 3	196	417	6	82	3	66	140	14	25	8
2	DMII_c cell 1	286	587	12	168	7	58	18	13	21	5
	DMII_c cell 2	275	724	4	199	3	94	39	12	23	6
	DMII_c cell 3	263	570	3	150	1	123	85	13	18	6
	DMII_c cell 4	252	622	11	157	7	58	16	12	13	6
3	DMII_d cell 1	146	325	16	47	5	36	27	10	3	8
	DMII_d cell 2	147	353	12	52	4	43	21	12	5	5
	DMII_d cell 3	150	367	12	55	4	43	24	12	4	5
	DMII_d cell 4	152	382	9	58	3	50	26	11	4	5
4	EMII_b cell 1	207	605	10	125	6	55	124	12	29	8
	EMII_b cell 2	212	725	7	153	5	68	125	12	27	8
	EMII_b cell 3	165	829	7	137	6	58	163	11	32	8
	EMII_b cell 4	183	628	16	115	10	41	140	13	43	7
5	EMII_c cell 1	253	587	8	148	5	68	30	13	11	7
	EMII_c cell 2	243	641	5	156	3	83	31	15	9	6
	EMII_c cell 3	251	654	8	164	5	67	32	13	17	6
	EMII_c cell 4	202	590	6	119	3	73	34	12	10	5
6	EMII_d cell 1	133	207	52	27	11	19	10	11	2	8
	EMII_d cell 2	101	185	65	19	12	15	10	13	3	8
	EMII_d cell 3	105	172	63	18	11	15	10	12	2	8
	EMII_d cell 4	96	193	61	19	12	15	9	11	4	7
7	PDMII_b cell 1	268	1243	10	333	12	64	214	12	34	9
	PDMII_b cell 2	221	1275	13	282	16	50	296	12	57	7
	PDMII_b cell 3	240	1255	10	301	12	60	239	14	37	9
	PDMII_b cell 4	204	1403	11	286	15	52	274	15	47	8
8	PDMII_c cell 1	157	314	23	49	7	31	20	11	18	6
	PDMII_c cell 2	172	355	35	61	12	27	23	11	29	7
	PDMII_c cell 3	173	314	22	54	7	33	21	12	13	7
	PDMII_c cell 4	170	377	16	64	6	39	20	11	20	6
9	PDMII_d cell 1	121	225	44	27	10	20	9	11	5	6
	PDMII_d cell 2	148	169	82	25	14	16	13	11	5	8
	PDMII_d cell 3	94	184	61	17	11	15	8	12	5	7

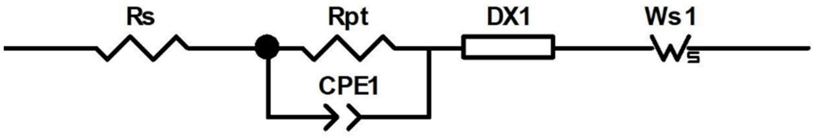

Figure S1. The equivalent circuit model used in the EIS study. This includes a series resistance (R_S), a resistance (R_{Pt}) and a constant phase element (CPE1) to model a counter electrode, an extended distributed element (DX1) to represent the mesoporous TiO_2/electrolyte interface as a transmission line model, and a Warburg element (Ws1), which represents the diffusion of the electrolyte.

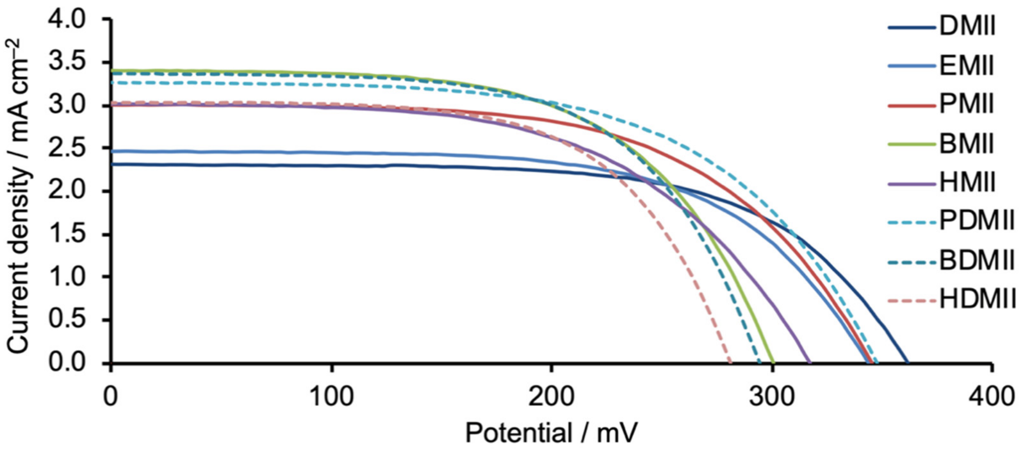

Figure S2. *J*–*V* curves for DSCs containing electrolytes with different ionic liquids (see Scheme 2 for abbreviations) and 0.05 M I_2 in the initial electrolyte.

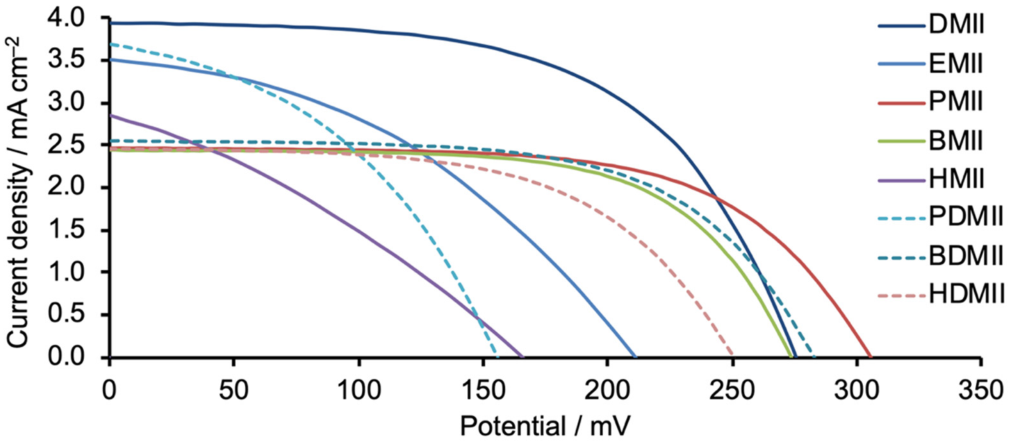

Figure S3. *J*–*V* curves for DSCs containing electrolytes with different ionic liquids (see Scheme 2 for abbreviations) and 0.10 M I_2 in the initial electrolyte.

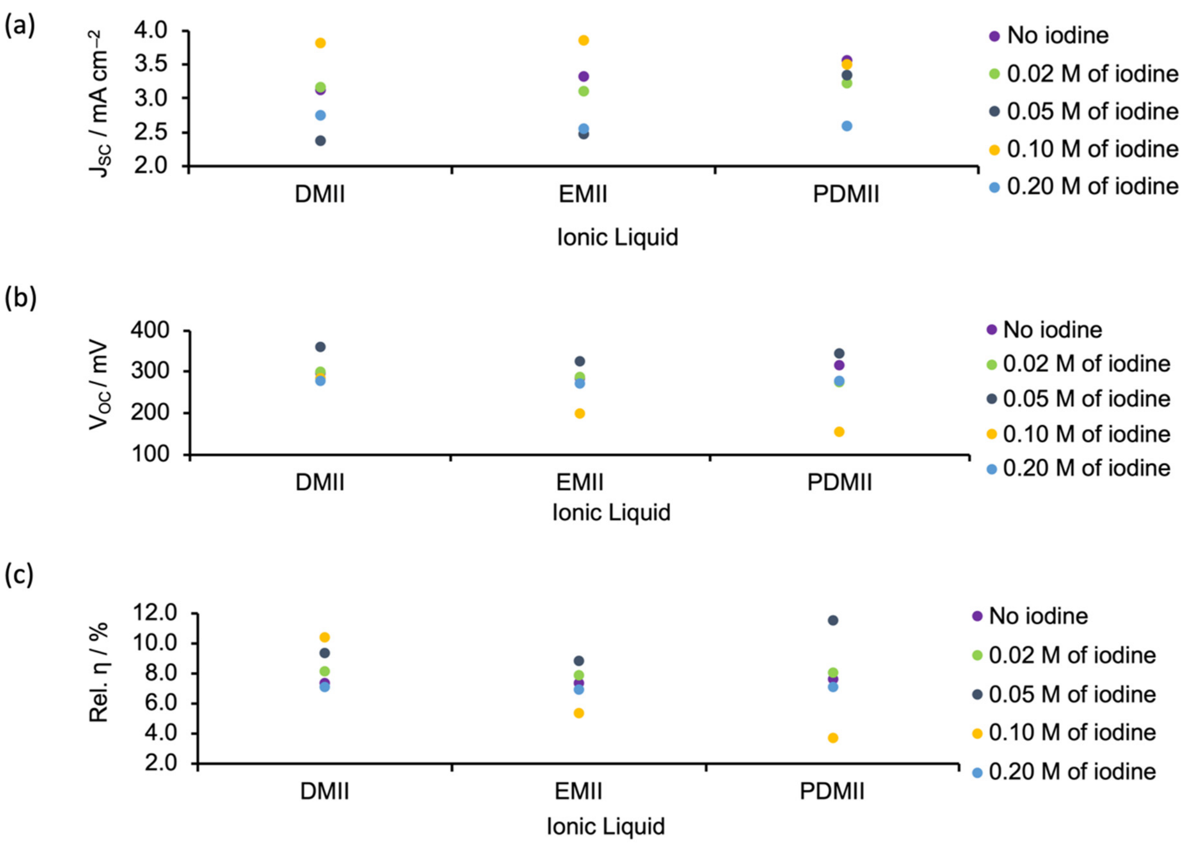

Figure S4. Average values of (**a**) J_{SC}, (**b**) V_{OC} and (**c**) η (relative to N719) for masked DSCs with different ILs and I_2 concentrations in the electrolytes.

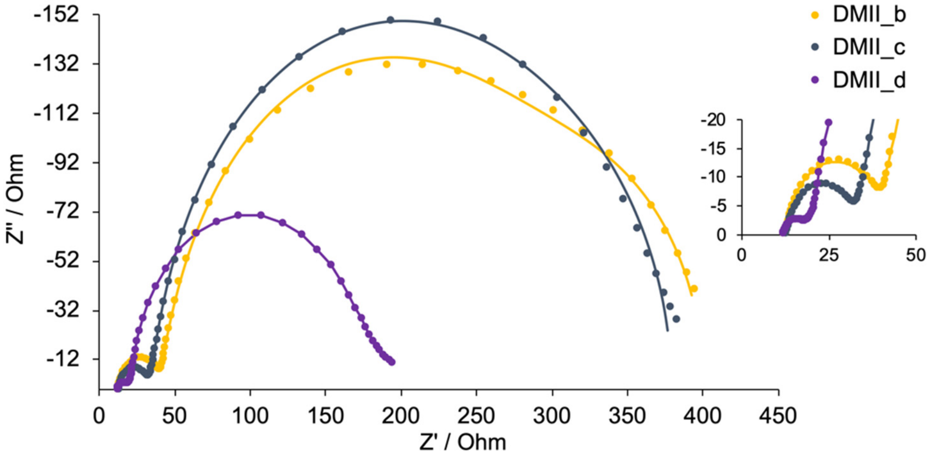

Figure S5. EIS Nyquist plots for DSCs with electrolytes with DMII IL. Solid lines represent fitted curves, and circle represent experimental data. The yellow colour corresponds to electrolytes with no added I_2, dark blue to electrolytes with 0.02 M I_2, and purple to 0.20 M I_2.

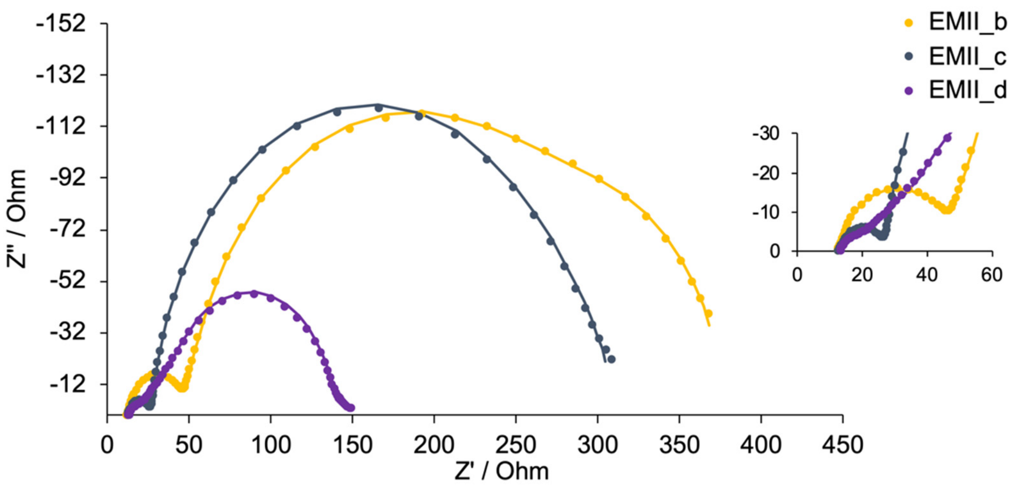

Figure S6. EIS Nyquist plots for DSCs with electrolytes with EMII IL. Solid lines represent fitted curves, and circle represent experimental data. The yellow colour corresponds to electrolytes with no added I_2, dark blue to electrolytes with 0.02 M I_2, and purple to 0.20 M I_2.

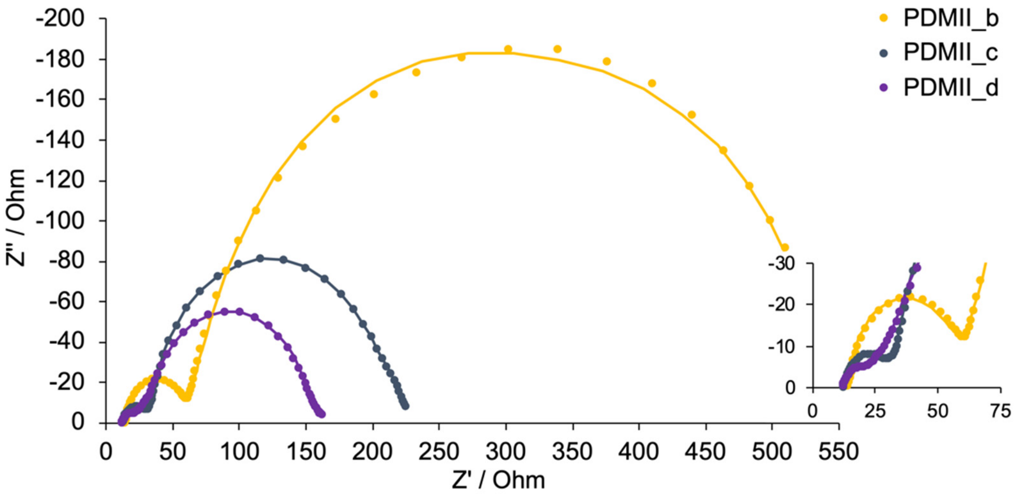

Figure S7. EIS Nyquist plots for DSCs with electrolytes with PDMII IL. Solid lines represent fitted curves, and circle represent experimental data. The yellow colour corresponds to electrolytes with no added I_2, dark blue to electrolytes with 0.02 M I_2, and purple to 0.20 M I_2.
